# Effect of diacerein on renal function and inflammatory cytokines in participants with type 2 diabetes mellitus and chronic kidney disease: A randomized controlled trial

**DOI:** 10.1371/journal.pone.0186554

**Published:** 2017-10-19

**Authors:** Fabiana Piovesan, Glaucia S. Tres, Leila B. Moreira, Michael E. Andrades, Hugo K. Lisboa, Sandra C. Fuchs

**Affiliations:** 1 Postgraduate Program in Cardiology, School of Medicine, Universidade Federal do Rio Grande do Sul (UFRGS), R. Ramiro Barcelos, Porto Alegre, RS, Brazil; 2 Hospital São Vicente de Paulo, School of Medicine, Universidade de Passo Fundo (UPF), R. Teixeira Soares, Passo Fundo, RS, Brazil; 3 Unidade de Análises Moleculares e de Proteínas (UAMP), Hospital de Clinicas de Porto Alegre, Universidade Federal do Rio Grande do Sul (UFRGS), R. Ramiro Barcelos, Porto Alegre, RS, Brazil; 4 Centro de Pesquisa Clinica (CPC), 5o. andar. Hospital de Clinicas de Porto Alegre, Universidade Federal do Rio Grande do Sul (UFRGS), R. Ramiro Barcelos, Porto Alegre, RS, Brazil; Universita degli Studi di Perugia, ITALY

## Abstract

Diacerein seems to improve metabolic control and reduce inflammatory marker levels in individuals with type 2 diabetes mellitus (Type 2 DM), but for participants with chronic kidney disease (CKD) its effect is unknown. This study aimed to evaluate the effect of diacerein *vs*. placebo on urinary albumin/creatinine ratio (ACR), glomerular filtration rate (GFR), and inflammatory cytokines in type 2 DM participants with CKD. Blood pressure (BP) and metabolic control were secondary outcomes. This randomized, placebo-controlled, parallel trial of adjuvant treatment of type 2 DM with diacerein enrolled seventy-two participants with CKD, aged 30–80 years, with glycated hemoglobin levels from 53–97 mmol/mol (7.0–11.0%), receiving angiotensin-converting enzyme inhibitors or angiotensin receptor blockers and antidiabetic agents. Participants randomized to diacerein or placebo were followed-up up to 90 days. Both groups had a marked reduction in ACR, but there was no effect on glomerular filtration rate. While the diacerein group had reduced TNF-α levels at the 75th percentile with a borderline significance (P = 0.05), there were no changes in the IL levels at the 75^th^ percentile.

Diacerein prevented the increase in blood glucose to the level observed in the placebo group (P = 0.04), improving metabolic control by 74%, reducing 24-hour diastolic BP, nighttime systolic and diastolic BP compared to the placebo group. In conclusion, among patients with type 2 DM and CKD, diacerein does not have an effect on ACR or GFR, but slows metabolic control deterioration and is associated with lower nighttime systolic and diastolic blood pressure.

**Trial registration:** Brazilian Clinical Trials Registry (Registro Brasileiro de Ensaios Clinicos; ReBeC) U1111-1156-0255

## Introduction

The growing global prevalence of type 2 diabetes mellitus (type 2 DM) has been accompanied by an increase in the burden of chronic kidney disease (CKD), which is estimated to affect around 50% of participants with type 2 DM worldwide [1]. A progressive decrease in glomerular filtration rate (GFR) and increased urinary albumin excretion [2] are among the pathophysiological mechanisms of CKD. In addition to metabolic, hemodynamic, and systemic changes, intrarenal inflammation has been recognized as playing a role in renal injury. Several studies have shown that increased levels of inflammatory markers, such as interleukin (IL)-1, IL-6, IL-18, tumor necrosis factor (TNF)-α, and transforming growth factor (TGF)-β, are independently associated with abnormal urinary albumin excretion [3–5].

In participants with Type 2 DM and nephropathy, inflammation has been considered as a potential mechanism explaining progressive renal function loss and current therapies are not sufficient [6–10] to prevent severe damage. Even though intensive treatment of diabetes delays the onset and slows the progression of CKD in insulin-treated type 2 DM participants [11], the current strategies are not sufficient to provide renal protection [12–13]. Thus, adjuvant therapeutic agents have been tested [14–20]. Among these, promising results have been obtained with diacerein, an anti-inflammatory drug used in the treatment of rheumatic disease. Diacerein inhibits the synthesis of interleukin, TNF-α, and proteases, and decreases the production of oxygen free radicals [21]. Experimental data [22–25] and results from a randomized controlled trial [26] on anti-diabetic naïve participants have shown improvement of metabolic control and levels of inflammatory markers in type 2 DM participants with diacerein treatment. Taking these aspects into consideration, the present trial was designed to evaluate the effect of diacerein *vs*. placebo on urinary albumin/creatinine ratio (ACR), GFR, and serum IL levels in participants with type 2 DM and CKD. As secondary outcomes, metabolic control of diabetes and blood pressure were evaluated.

## Material and methods

### Study design

In this randomized, placebo-controlled, parallel-group trial, participants were randomly assigned to receive diacerein (50 mg twice daily) or placebo for 90 days. Participants, physicians, and investigators were blinded to treatment allocation.

### Participants

Participants were recruited among Type 2 DM participants with CKD receiving care at the outpatient clinic of a tertiary hospital in Passo Fundo, southern Brazil, from January 29 to April 4^th^ 2014, and the follow up was completed on June 20, 2014. Eligible participants included men and women aged 18 to 80 years with a diagnosis of Type 2 DM, receiving antidiabetic medication, and with CKD (ACR ≥300 mg/g or GFR between 30 and 100 mL/min/1.73 m^2^). All participants were using an angiotensin converting enzyme (ACE) inhibitor or angiotensin receptor blocker (ARB) and had A1C levels between 7.0 and 11.0%. Exclusion criteria were body mass index (BMI) ≥40 kg/m^2^, pregnancy or breastfeeding, rheumatic diseases, cancer, previous pancreatitis, hypersensitivity to rhein, or severe liver or gastrointestinal disease. The authors confirm that all ongoing and related trials for this drug/intervention were registered. Firstly, the study protocol was registered in the Plataforma Brasil, a condition to be submitted to the Ethics Committee. It was approved by the Ethics Committee of the Hospital de Clinicas de Porto Alegre (GPPG number 120482), which is accredited by the Office of Human Research Protections as an Institutional Review Board, on January 7, 2013. After that, the trial was registered in the Brazilian Clinical Trials Registry (Registro Brasileiro de Ensaios Clinicos (ReBeC), under the number: U1111-1156-0255) (Data in S1 Text and S2 Text). A written informed consent was obtained from all participants, according to the principles expressed in the Declaration of Helsinki. The trial was designed, implemented, and described following the CONSORT statement (Data in S3 Text).

### Randomization, allocation concealment, and blinding

The randomization sequence was generated at the coordinating center, before the start of the trial, using an automated web-based software (Random Allocation Software). Allocation was performed by block randomization with permuted block sizes of four and six and allocation ratio of 1:1. In order to ensure concealment of the allocation list, randomization was implemented through a 24-hour web-based automated randomization system (Research Electronic Data Capture, REDCap) and alphanumeric codes were used to keep the investigators, participants, and physicians blinded to the allocation. Blinding was maintained until the end of the trial.

### Intervention

Participants received diacerein (50 mg capsules twice daily, every 12 hours) or placebo on the same schedule. Placebo, an inert substance, had an identical-looking packaging and both, Diacerein and placebo, were manufactured by a pharmaceutical company (TRB Pharma, Campinas, SP, Brazil) and provided free of charge to all participants. The study drugs were dispensed by a pharmacist at the clinical center. Participants returned for clinical visits seven days after randomization in order to assess adverse effects that prevented adherence to treatment, at 30 and 60 days to receive a new supply of capsules, and at 90 days for evaluation of primary and secondary outcomes. Participants were requested not to change lifestyle, food intake, and current medication.

### Study procedures

Potentially eligible participants underwent clinical and laboratory evaluation in three consecutive clinical visits, held in the morning, in order to confirm inclusion criteria. Laboratory measurements, including A1C, fasting serum glucose, fasting insulin, creatinine, IL-1β, IL-6, IL-8, IL-10, TNF-α, adiponectin, leptin, selectin, and urinary albumin excretion (morning sample spot) were obtained at enrollment and again at the final follow-up visit. Spot urine samples were used to determine albumin and creatinine concentrations and to calculate the ACR. In addition, ACR was categorized as < 300 or ≥300 mg/g. GFR was calculated using the Chronic Kidney Disease Epidemiology Collaboration (CKD-EPI) equation [27], which has advantages over the Modification of Diet in Renal Disease (MDRD) equation in terms of accuracy [28]. Insulin resistance was determined by the homeostasis model assessment of insulin resistance (HOMA-IR) index. Fasting glucose was measured by an enzymatic and colorimetric method, creatinine by the Jaffe method, insulin by enzyme-linked immunosorbent assay (ELISA), and A1C and albuminuria were determined by immunoturbidimetric assay. Interleukin levels were measured using the Luminex platform (immunofluorescence assay), while serum selectin, leptin, and adiponectin levels were measured by ELISA (Invitrogen kit) in duplicate samples.

The follow-up clinical visits aimed to verify adherence to treatment, investigate adverse events, and provide participants with the study drugs. Adherence to treatment was defined as a ratio ≥ 80% between the number of returned capsules over the capsules dispensed. Adverse events, particularly the occurrence of nausea, vomiting, diarrhea, loose stools, abdominal pain, pruritus, and dark urine, were investigated using a semi-structured questionnaire. Open ended questions were also used to elicit information about other adverse events.

Standardized measurement of office blood pressure, performed in duplicate, was carried out at baseline and at the end of the trial, using an oscillometric monitor (MICROLIFE). The mean of two measurements was used for analysis. In addition, 24-hour ambulatory blood pressure monitoring (ABPM) was performed using a Cardioserv monitor (DYNAMAP model, version 2007) at baseline and at the end of the trial. All data were entered into a REDCap electronic form.

### Outcomes

The primary outcomes were reduction ≥ 15% in ACR, any reduction in GFR, improvement of lack of metabolic control (defined as A1C > 7% and fasting glucose > 126 mg/dL), reduction in plasma levels of IL-1, IL-6, IL-8, and TNF-α, increase in plasma levels of IL-10, and reduction in blood pressure. Additionally, serum levels of fasting insulin, adiponectin, leptin, and selectin were analyzed.

### Sample size calculation and statistical analysis

The sample size calculation was based on estimates, since there was no previous data on the reduction of ACR using diacerein among participants with CKD. The cut points for mild CKD of 30 mg/g to moderate CKD of 45 mg/g were used as the standard deviations (SD) of A/C rate in both groups. Sample size was estimated for reductions between intervention and placebo groups ranging from 20 to 40 mg/g. Approximately, 72 participants were required to detect a reduction in ACR of at least 20 mg/g ±30 mg/g to 30 mg/g ±45 mg/g in participants using diacerein, with 80% power and P (alpha) of 0.05, considering a 1:1 ratio of intervention to control participants.

Trial results were analyzed using the intention-to-treat approach. For continuous variables, the assumptions to use t-tests were verified using the Shapiro-Wilks test (for normal distribution) and the Levene’s test (for homogeneity of variance) and equal variances were assumed. Therefore, baseline characteristics were analyzed using the t test for independent samples, chi-square test for categorical variables, HOMA and urinary albumin/creatinine ratio (mg/g) were analyzed using Mann-Withney to compare median and inter-quartile range (md; IQR: 25–75). Generalized Estimating Equations (GEE) models were used to analyze the group, time, and time*group differences. Moreover, the normal and gamma distributions were tested with covariance matrix with a robust estimator in an unstructured and exchangeable structures. For each variable, the model was selected using the Quasi-Akaike Information Criterion (QIC) of the goodness-of-fit. The lack of metabolic control (yes or no) was analyzed as outcome, while controlling for the lack of metabolic control in the baseline, using a binomial logistic regression. The estimated relative risk (Odds ratio, OR) was used to determine the corresponding relative risk reduction (RRR = 1-OR). The goodness of fit was described by the Hosmer and Lemeshow test, the percentage of cases correctly classified, and the Nagelkerke *R*^*2*^—to explain variation in the dependent variable based on the model with the intervention and lack of metabolic control at the baseline. Inflammatory markers were categorized at the 75^th^ percentile of values obtained at baseline. For one participant of the diacerein group, the last observed value of lab tests was carried to the end of the trial. A *P*-value < 0.05 was considered to be statistically significant and a 0.05 < *P*-value < 0.15 was considered a trend toward association. Because the distribution of participants with ACR ≥300 was unbalanced between the groups at baseline, an initially unplanned exploratory analysis of ACR *vs*. treatment group stratified by ACR ≥300 mg/g was carried out. All analyses were performed using SPSS for Windows (version 17.0; SPSS Inc., Chicago, IL, USA).

## Results and discussion

**Fig 1:**
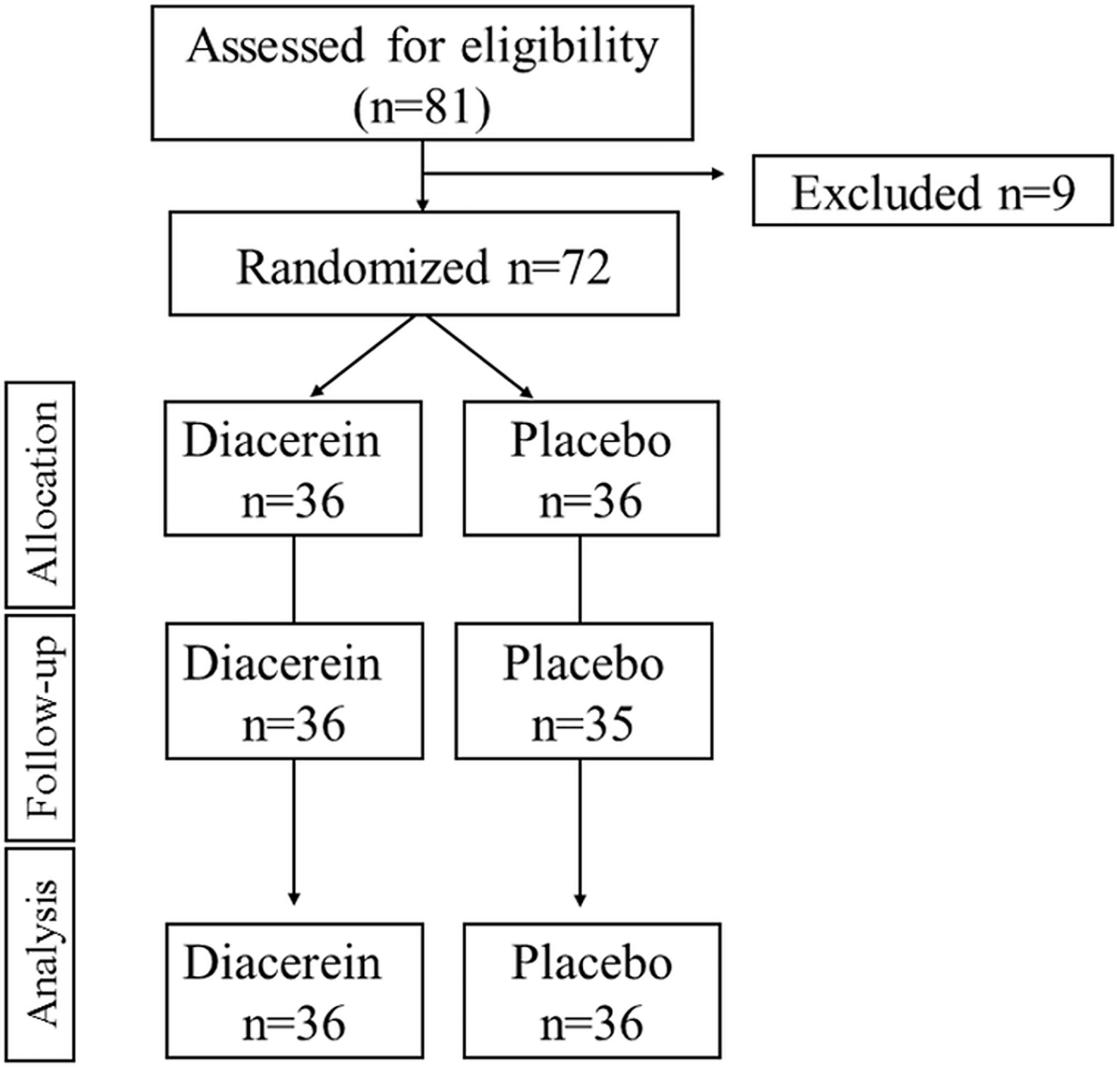
Flow diagram of participant selection, randomization, and follow-up.

From September 2013 to March 2014, 81 individuals with CKD and Type 2 DM were assessed for eligibility. Of these, 72 met the inclusion criteria and were randomly assigned to receive diacerein (*n* = 36) or placebo (*n* = 36) (Fig 1). At the end of the trial, 36 placebo-treated and 35 diacerein-treated participants were evaluated. One participant in the diacerein group was lost to follow-up because of family migration. The anonymized dataset is uploaded as a Supporting Information file (S1 File).

The baseline characteristics of the participants are shown in Table 1. Mean (SD) participant age was 61.6 (11.0) years. There were no marked differences between the two groups regarding age, gender distribution, years at school, smoking, hypertension, and duration of Type 2 DM. A higher number of placebo participants had diabetic foot and ACR ≥300 mg/g. Insulin and ACE inhibitor use and GFR were higher in the diacerein group. The mean adherence rate was similar between participants in the diacerein (83%) and in the placebo group (79%).

**Table 1:** Baseline characteristics of participants according treatment group [Mean ± SD or n (%) or median (interquartile range)].

Characteristic	Diacerein (*n* = 36)	Placebo (*n* = 36)	*P*
**Age (years)**	60.6 ±11.5	62.5 ±10.1	0.5
**Male gender**	19 (52.8)	23 (63.9)	0.3
**Years at school**	7.9 ±4.6	7.1 ±4.2	0.5
**Current or past smoking**	15 (41.7)	12 (33.3)	0.5
**Systolic blood pressure (mmHg)**	130.1 ±12.7	135.8±17.1	0.12
**Diastolic blood pressure (mmHg)**	74.5 ±10.3	78.3 ±10.8	0.13
**Hypertension**	20 (55.6)	19 (52.8)	0.8
**Duration of diabetes (years)**	13.3 ±8.7	13.4 ±7.3	0.9
**Diabetic foot**	2 (5.6)	5 (13.9)	0.2
**Fasting glucose (mg/dL)**	145.4 ±68.3	141.1 ±75.0	0.8
**Glycated hemoglobin (%)**	9.0 ±1.2	8.9±1.2	0.6
**Lack of metabolic control**	23 (63.9)	19 (52.8)	0.3
HOMA-IR*	4.5 (3.1–7.0)	3.7 (2.6–10.7)	0.7
Urinary albumin/creatinine ratio (mg/g)*	80.1 (37.5–162.2)	92.9 (47.8–363.2)	0.3
**Urinary albumin/creatinine ≥ 300 mg/g**	5 (13.9)	9 (25.0)	0.2
**Glomerular filtration rate (mL/min/1.73 m**^**2**^**)**	70.5 ±21.1	63.1 ±19.5	0.13
**Creatinine (mg/dL)**	1.1 ±0.3	1.2 ±0.3	0.06
Use of oral hypoglycemic agents**			
** Sulfonylureas**	11 (30.6)	16 (44.4)	0.22
** Biguanides**	27 (75.0)	33 (91.7)	0.06
**Other antidiabetic drugs**	4 (11.1)	4 (11.1)	1.0
**Insulins**	25 (69.4)	19 (52.8)	0.15
**Lipid-lowering agents**	25 (69.4)	34 (94.4)	0.06
Antihypertensive drugs**			
** Angiotensin-converting enzyme inhibitor**	26 (72.2)	20 (55.6)	0.14
** Angiotensin receptor blocker**	12 (33.3)	19 (52.8)	0.10

HOMA-IR = Homeostasis Model Assessment of Insulin Resistance

* Analysis using Median (Md) and interquartile range (IQR: 25–75)

** Numbers exceed 100% due to simultaneous use of more than one agent.

As shown in Table 2, a marked reduction in ACR was detected in both treatment groups, but there was no statistical significance difference among groups. GFR remained similar from baseline to the end of the trial, and there was no interaction of time and group. A lower mean increase in fasting glucose levels was observed in the diacerein group as compared to the placebo group (P = 0.04). Regarding lack of metabolic control, at the end of the trial 29% participants in the diacerein group had A1C > 7% and fasting glucose > 126 mg/dL (*vs*. 46%) in the placebo group (P = 0.03). The binomial logistic regression model was statistically significant, χ^2^(2) = 24.727; *P*< 0.0001, had a Hosmer and Lemeshow test with a P value = 0.964, a Nagelkerke *R*^*2*^ explaining 38.8% of the variance in lack of metabolic control, and had correctly classified 75.0% of cases. Participants in the diacerein group were protected of lack of metabolic control (OR = 0.259 (95%CI: 0.075–0.896; P = 0.03), in comparison with placebo and independently of fasting glucose and glycated hemoglobin at the baseline, which resulted in an overall relative reduction of 74.1% of lack of metabolic control with administration of diacerein. Nighttime systolic and diastolic blood pressure were reduced in participants treated with diacerein and increased in the placebo group.

**Table 2:** Effect of diacerein versus placebo on changes in renal function markers, control of type 2 diabetes, and blood pressure (mean ±SE).

Variable	Group	Baseline (*n* = 36)	End of trial (*n* = 36)	P*	
Urinary Albumin/ creatinine ratio (mg/g)‡	Diacerein	84.7 ±1.3	63.9 ±1.2	0.3	
	Placebo	118.0 ± 1.3	61.1 ±1.2		
Glomerular filtration rate (mL/min/1.73 m^2^)	Diacerein	70.5 ±3.5	70.1 ±3.7	0.3	
	Placebo	63.1 ±3.2	64.9 ±3.46		
Creatinine (mg/dL)	Diacerein	1.0 ±0.05	1.1 ±0.05	0.3	
	Placebo	1.2 ±0.05	1.1 ±0.06		
Glycated hemoglobin (%)	Diacerein	9.0 ±0.2	8. ±0.3	0.3	
	Placebo	8.9 ±0.2	8.5 ±0.3		
Fasting glucose (mg/dL)	Diacerein	145.4 ±11.2	156.4 ±13.2	0.03	
	Placebo	141.1 ±12.3	186.3 ±14.9		
HOMA-IR‡	Diacerein	84.7 ±1.1	85.1 ±1.2	0.4	
	Placebo	5.1 ±1.2	6.7 ±1.2		
24-h SBP (mmHg)	Diacerein	129.9 ±2.5	132.6 ±2.9	0.13	
	Placebo	127.2 ±2.3	134.8 ±2.9		
24-h DBP (mmHg)	Diacerein	77.3 ±1.6	76.4 ±1.5	0.03	
	Placebo	79.1 ±1.7	82.0 ±1.8		
Daytime SBP (mmHg)	Diacerein	129.4 ±2.4	134.4 ±2.8	0.4	
	Placebo	127.6 ±2.3	135.5 ±2.8		
Daytime DBP (mmHg)	Diacerein	78.0 ±1.0	78.2 ±1.4	0.05	
	Placebo	78.3 ±2.6	83.6 ±1.8		
Nighttime SBP (mmHg)	Diacerein	131.1 ±3.0	128.2 ±3.2	0.009	
	Placebo	126.0 ±2.8	132.0 ±3.5		
Nighttime DBP (mmHg)	Diacerein	75.1 ±1.7	71.8 ±1.7	0.03	
	Placebo	76.1 ±2.0	77.8 ±2.1		

SBP = systolic blood pressure; DBP = diastolic blood pressure

HOMA-IR = Homeostasis Model Assessment of Insulin Resistance

* Analysis using GEE, with gamma distribution and exchangeable correlation matrix, showing P value for interaction group*time

‡ Urinary Albumin/ creatinine ratio and HOMA-IR were transformed (natural logarithm) for analysis and the results were exponentiated

**Fig 2:**
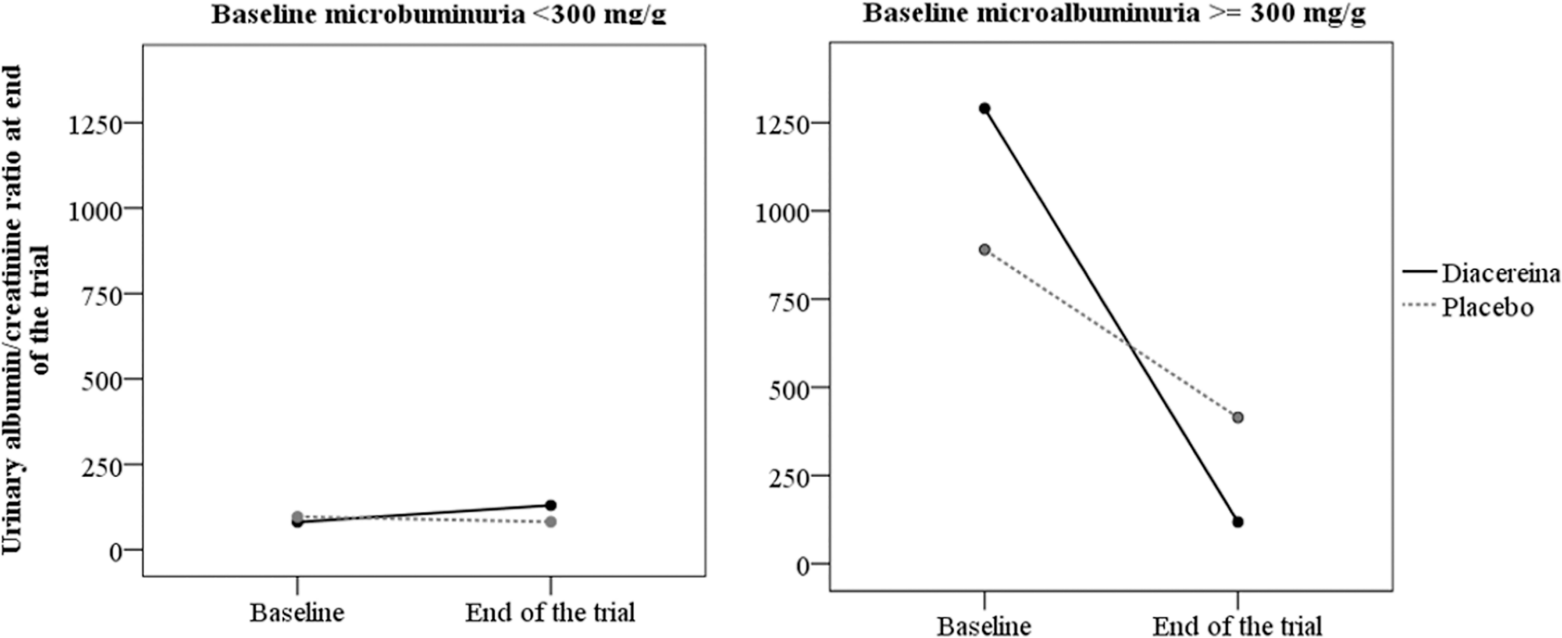
End-of-trial changes in ACR in diacerein and placebo participants stratified by baseline ACR ≥300 or < 300 mg/g. Interaction of time and group (*P* = 0.006).

The exploratory analysis of ACR changes between groups in participants stratified by ACR ≥300 mg/g is shown in Fig 2. At end of the trial, diacerein participants with baseline ACR ≥300 mg/g had greater reduction of ACR than placebo participants (*P* for interaction = 0.006). There were no differences in ACR variation between diacerein and placebo participants with ACR < 300 mg/g.

**Fig 3:**
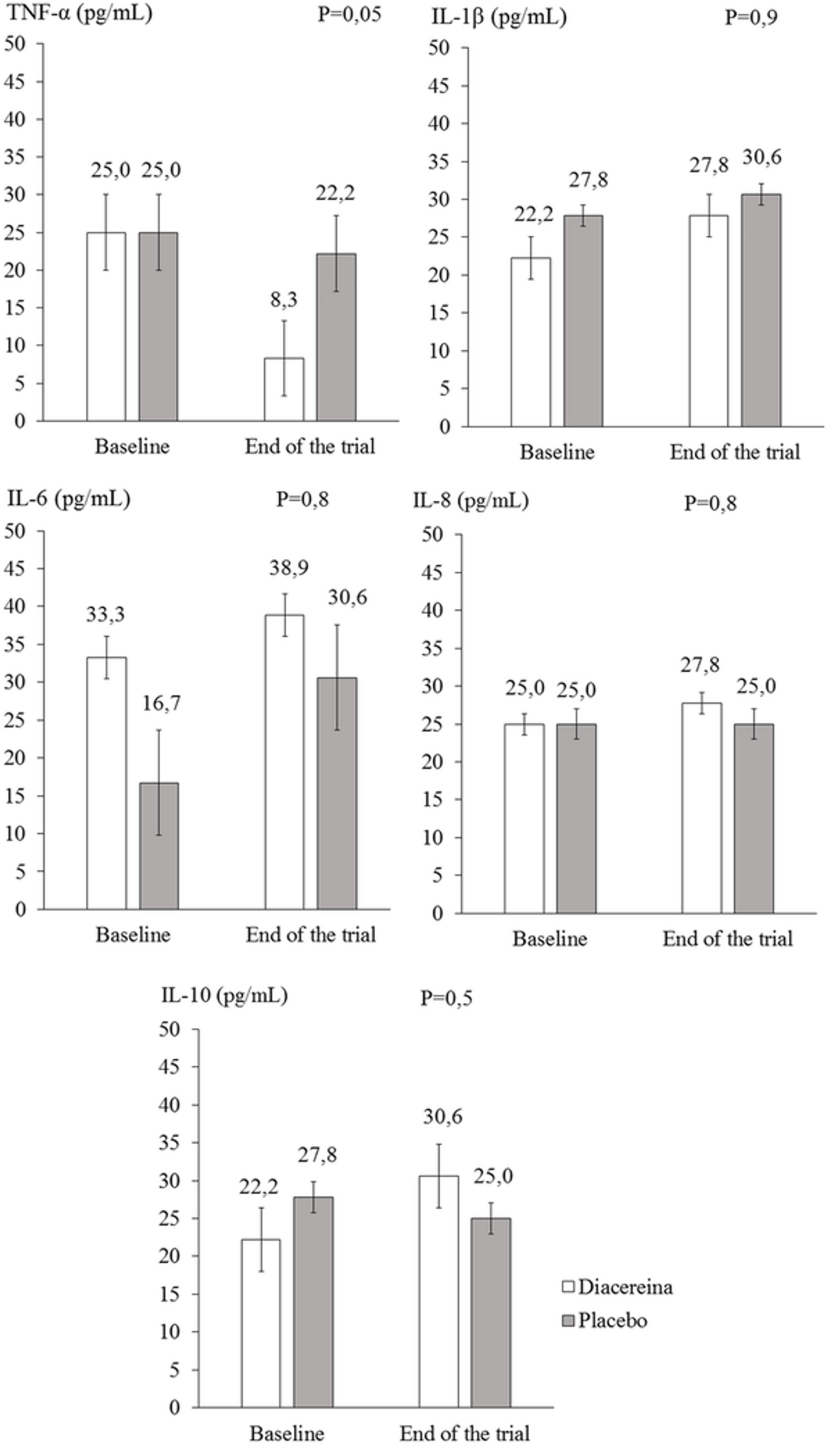
Levels of inflammatory markers in diacerein and placebo groups at baseline and end of trial.

There were no significant end-of-trial differences between the diacerein and placebo groups in the IL levels at the 75^th^ percentile. As shown in Fig 3, the comparison between the groups regarding TNF-α levels reached borderline significance (*P* = 0.05). No significant changes were observed in adiponectin, leptin, and selectin levels within or between the groups after 90 days of diacerein/placebo treatment (Table 3).

**Table 3:** Changes in adipokines according to randomization group (mean ± SE).

Adipokine	Group	Baseline	End of trial	P*
Adiponectin (ng/mL)	Diacerein	18.0 ±1.6	18.1 ±1.5	0.8
	Placebo	17.2 ±1.4	17.7 ±1.4	
Leptin (ng/mL)	Diacerein	25.1 ±3.9	21.4 ±2.7	0.5
	Placebo	25.6 ±2.8	24.4 ±2.9	
Selectin (ng/mL)	Diacerein	52.2 ± 5.0	47.7 ±4.9	0.7
	Placebo	58.3 ± 6.1	56.7 ±6.5	

* Analysis using GEE, with gamma distribution and exchangeable correlation matrix, showing P value for interaction group*time

Regarding adverse events, there was no statistically significant difference in self-reported global or individual adverse events among treatment groups, in addition to dark urine and loose stools (S1 Table). Participants of the diacerein group were more likely to report dark urine than those of the control group (P = 0.003), as well as loose stools (P = 0.004). Abdominal pain was the adverse events more frequently cited by participants of diacerein arm, while diarrhea was often and similarly reported by participants of both groups. Vomiting and pruritus were reported by 2 (5.6%) participants allocated to diacerein and none allocated to placebo (P = 0.15). No participant allocated to the diacerein group discontinued his/her participation in the study due to an adverse event.

This randomized controlled trial was the first to evaluate the effect of diacerein on parameters of renal function and inflammatory markers among type 2 DM participants with CKD. Outpatients of a reference center may not have marked excretion of microalbuminuria, and yet, the question is: diacerein helps reduce incipient microalbuminuria? The results show that diacerein had no effect on GFR and ACR, but might led to improvement in metabolic control and reduction in nighttime systolic and diastolic blood pressure in type 2 DM. In addition, exploratory analysis detected a subgroup (ACR ≥300 mg/g) of participants who responded to diacerein treatment with a decrease in ACR, a finding which deserves evaluation in future studies. Therefore, hypothesis tests for primary objective were negative, but a positive result has emerged for an exploratory analysis of ACR and only for the inflammatory marker–TNF-α. Long-term renal involvement may be unresponsive to administration of diacerein for 90 days, or, conversely, a 90-day period might have been insufficient to produce an effect on participants with a lower degree of renal impairment.

As an adjunctive agent in the treatment of diabetes, diacerein was effective in improving metabolic control of diabetes, reducing the proportion of participants with abnormal levels of both blood glucose and A1C comparatively to placebo group. These results are consistent with those of the only previous randomized controlled trial of diacerein involving type 2 DM participants to date [26]. However, there are striking differences between the two trials. First, in the previous study, recruitment was limited to participants with less than six months since diagnosis and not receiving antidiabetic treatment, whereas we enrolled type 2 DM participants with established kidney disease using antidiabetic medication. Further, all 72 participants in the present study underwent a 90-day treatment course with diacerein or placebo; in the previous study, in addition to the smaller number of participants (40 participants), using escalated doses of diacerein, administered for 60 rather than 90 days. Nevertheless, in both trials, diacerein was equally able to induce a significant decrease in fasting glucose and A1C levels, an effect possibly resulting from improved insulin secretion, since no changes were observed in insulin sensitivity.

At baseline, diacerein-treated participants showed a trend (P < 0.15) toward lower systolic and diastolic blood pressures compared to placebo participants, which is consistent with more frequent use of ACE inhibitors. Regardless, during follow-up, nighttime systolic and diastolic blood pressures were reduced in the diacerein group and increased in the placebo group. This adjuvant hypotensive effect of diacerein cannot be explained by changes in antihypertensive treatment, since participants did not change blood pressure lowering medication during the trial.

In the present trial, diacerein also had an effect on TNF-α levels. Even though the changes in TNF-α levels had borderline significance, they are consistent with the observations of the previous trial of diacerein in type 2 DM participants [26]. Because TNF-α plays a role in the induction of the cytokine cascade, reduction of this polypeptide could predict an effect of diacerein on other cytokines. Changes in C-Reactive Protein (CRP) were also evaluated (non-ultra-sensitive CRP), but the results were not statistically significant (S2 Table). Additionally, differences were found between the diacerein and placebo groups regarding use of lipid-lowering agents and biguanides, which was more frequent in the placebo group; this may have attenuated the inflammatory process, thus minimizing the differences between groups. The experimental basis for the efficacy of diacerein derives originally from experiments performed in type 2 DM mice, in which diacerein reduced subclinical chronic inflammation at the cellular level in the liver, adipose tissue, and muscle, in addition to reducing hepatic glucose production [22,23]. The mechanisms underlying the benefits observed with the use of diacerein probably result from control of intrarenal inflammation, with reduced interleukin production and improved metabolic (glycemic) control, and reduction of nighttime blood pressure, thereby curtailing renal damage. However, the lack of effect of diacerein on inflammatory markers does not preclude the possibility that a longer intervention might modify these results.

The effects on metabolic control of diabetes and nighttime blood pressure associated with the number of participants reporting adverse events in the diacerein group indicate that the drug was well tolerated. Dark urine and loose stools are mild adverse events that probably would not prevent its use. Therefore, the present study provides data to add evidence in favor of the use of diacerein as an adjuvant therapy by patients with type 2 DM and renal disease. However, there is a need for more information to consolidate its potential benefits.

## Conclusions

In conclusion, diacerein treatment of type 2 DM participants with established kidney disease has no effect on GFR and ACR, but may lead to improved metabolic control of diabetes, reduction of nighttime BP, and possibly reduction of TNF-α levels. Further studies including larger number of participants exposed to long-term treatment with diacerein are warranted to confirm these findings.

## Supporting information

S1 Table Frequency of adverse events according to treatment groups [n (%)]. (DOCX)

S2 Table Analysis of C-Reactive Protein at the baseline and the end of trial among diacerein and placebo groups [(md; IQR: 25–75)]. * P value for analysis between CRP at the baseline and end of trial.
** P value for analysis of CRP at the end of trial among diacerein *vs*. placebo.
*** Analysis of median; interquartile range (Md; IQR: 25–75) using Mann-Whitney test.
(DOCX)

S1 Text Protocol of diacerein in English. (PDF)

S2 Text Protocol of diacerein in Portuguese. (PDF)

S3 Text CONSORT 2010 checklist of information to include when reporting a randomised trial. (DOC)

S1 File Diacerein PLOSONE dataset file. (XLSX)
