# Supplementary material for: Effect of diacerein on renal function and inflammatory cytokines in participants with type 2 diabetes mellitus and chronic kidney disease: A randomized controlled trial
Source: PLoS One. 2017 Oct 19;12(10):e0186554. doi: 10.1371/journal.pone.0186554 (PMC5648185; doi:10.1371/journal.pone.0186554)
Supplement: S1 Table — (DOCX) [file pone.0186554.s001.docx]

**S1 Table. Frequency of adverse events according to treatment groups [n (%)]**

| **Adverse events** | **Diacerein (*n*= 36)** | **Placebo (*n*= 36)** | **P** |
| --- | --- | --- | --- |
| Nausea | 2 (5.6) | 1 (2.8) | 0.6 |
| Vomiting | 2 (5.6) | 0 | 0.15 |
| Diarrhea | 3 (8.3) | 3 (8.3) | 1.0 |
| Loose stools | 4 (11.1) | 0 | 0.04 |
| Abdominal pain | 3 (8.3) | 1 (2.8) | 0.3 |
| Dark urine | 8 (22.2) | 0 | 0.003 |
| Pruritus | 2 (5.6) | 0 | 0.15 |
| Other | 1 (2.8) | 5 (13.9) | 0.10 |
| Total number of symptoms |  |  | 0.4 |
| 0 | 21 (58.3) | 27 (75.0) |  |
| 1 | 11 (30.6) | 8 (22.2) |  |
| 2 | 1 (2.8) | 1 (2.8) |  |
| 3 | 2 (5.6) | 0 |  |
| 4 | 1 (2.8) | 0 |  |
