# Supplementary material for: Effect of diacerein on renal function and inflammatory cytokines in participants with type 2 diabetes mellitus and chronic kidney disease: A randomized controlled trial
Source: PLoS One. 2017 Oct 19;12(10):e0186554. doi: 10.1371/journal.pone.0186554 (PMC5648185; doi:10.1371/journal.pone.0186554)
Supplement: S2 Table — * P value for analysis between CRP at the baseline and end of trial. ** P value for analysis of CRP at the end of trial among diacerein vs. placebo. *** Analysis of median; interquartile range (Md; IQR: 25–75) using Mann-Whitney test. (DOCX) [file pone.0186554.s002.docx]

S2 Table.Analysis of C-Reactive Protein at the baseline and the end of trial among diacerein and placebo groups [(md; IQR: 25-75)]

| **Variable** | **Group** | **Baseline (*n*= 36)** | **End of trial (*n*= 36)** | ***P**** | ***P***** |
| --- | --- | --- | --- | --- | --- |
| CRP (mg/g)******* | Diacerein | 5.0 (2.0-10.9) | 4.8 (2.7-11.4) | 0.6 | 0.4 |
|  | Placebo | 3.3 (1.9-7.9) | 4.1 (2.6-10.0) | 0.2 |  |

S2 Table legend.

* P value for analysis between CRP at the baseline and end of trial
 ** P value for analysis of CRP at the end of trial among diacerein *vs*. placebo

*** Analysis of median; interquartile range (Md; IQR: 25-75) using Mann-Whitney test
