# Supplementary material for: Effect of diacerein on renal function and inflammatory cytokines in participants with type 2 diabetes mellitus and chronic kidney disease: A randomized controlled trial
Source: PLoS One. 2017 Oct 19;12(10):e0186554. doi: 10.1371/journal.pone.0186554 (PMC5648185; doi:10.1371/journal.pone.0186554)
Supplement: S1 Text — (PDF) [file pone.0186554.s003.pdf]

PROTOCOL

EFFECT OF DIACEREIN ON RENAL FUNCTION AND INFLAMMATORY CYTOKINES IN PARTICIPANTS WITH TYPE 2 DIABETES MELLITUS AND CHRONIC KIDNEY DISEASE: A RANDOMIZED CONTROLLED TRIAL

Authors
Fabiana Piovesan, Gláucia S. Três, Leila B. Moreira, Michael Andrades, Hugo K. Lisboa, Sandra C. Fuchs

Registration
Plataforma Brasil/Sistema Nacional de Ética em Pesquisa
CAAE: 07049612.2.0000.5327
Ethics Committee of the Hospital de Clinicas de Porto Alegre (GPPG number120482)

Institutions/ sponsors
Hospital de Clinicas de Porto Alegre, Universidade Federal do Rio Grande do Sul (UFRGS), Hospital São Vicente de Paulo, CNPq

Health conditions
Diabetes mellitus
Diabetic nephropathy
Chronic kidney disease

BACKGROUND

Diabetes mellitus is a chronic disease that can cause micro and macrovascular complications generating relevant morbidity and mortality (1,4,5). It is estimated that approximately 8.2% of adults are affected and, by 2030, at about 366 million of individuals will have diabetes (2,3). Diabetic nephropathy (DN) is one of the complications of type 2 diabetes mellitus (T2DM), occurring in 20-40% of diabetic patients, and is the main cause of chronic dialytic kidney disease in developed countries (1). DN is determined by increased urinary albumin excretion (microalbuminuria or macroalbuminuria) and / or reduced glomerular filtration rate (GFR) (6,7,8).

The advance of the knowledge on pathophysiology of diabetic nephropathy through molecular and genetic analyzes point to the immune system as a mediator and also

as responsible for complications of the disease. Inflammatory cytokines, especially interleukin-1 (IL-1), interleukin-6 (IL-6) and interleukin-18 (IL-18) as well as tumor necrosis factor (TNFα), would participate in the development and progression of diabetic nephropathy. The presence of mast cells in the tubular intersticium with release of chymase, transforming growth factor (TGF-β1), renin and TNF-α would lead to renal structural damage as well as inflammation resulting, ultimately, in fibrosis (9,10). Despite the benefits of renoprotective medications, such as angiotensin receptor blocker and angiotensin converting enzyme inhibitor, the high prevalence of diabetic nephropathy characterizes the need for new therapeutic agents.

The potential to affect the primary mechanisms of DN led to the identification and testing of new agents, for example, to reduce proteinuria caused by diabetes, such as statins, pentoxifylline, mycophenolate mofetil, endothelin receptor antagonist, proteinase kinase C inhibitor, fenofibrates and fish oil (11,12,13). A randomized clinical trial in diabetic subjects demonstrated a reduction on fasting glycemia, glycated hemoglobin, and inflammatory markers after administration of diacerein, an anthraquinone derivative with anti-inflammatory properties (14,15). Other therapies focused on the inflammatory process of diabetic nephropathy seem to be necessary to promote remission or even regression of renal injury. Since diacerein has anti-inflammatory properties and has already shown an effect in patients with DM (14), we believe that its effect should be investigated in patients with renal impairment due to diabetes mellitus.

RESEARCH QUESTIONS

1.Does diacerein improve diabetic nephropathy in subjects with type 2 diabetes mellitus, assessed by the urinary albumin / creatinine ratio and GFR?

2.Does diacerein modify serum levels of inflammatory cytokines in subjects with type 2 diabetes mellitus assessed by the measurement of IL-1β, IL-6, IL-8, IL-10 and TNF-α?

3.Does diacerein improve metabolic control and blood pressure in subjects with type 2 diabetes mellitus with diabetic nephropathy?

METHODS

Study design

This is a double-blind, randomized controlled trial with two-arm, a phase 3 controlled trial.

Eligibility criteria

This trial will include men and women aged 30 to 80 years, with diagnosis of type 2 DM, in regular use of antidiabetic medication - angiotensin converting enzyme inhibitor or angiotensin receptor blocker, with body mass index <40kg/m², and CKD (ACR $\geq$ 300 mg/g or GFR between 30 and 100 mL/min/1.73 m^2), with A1C levels between 7.0 and 11.0%, using antidiabetic drugs, who accept to participate and sign free and informed consent.

Exclusion criteria

Exclusion criteria include pregnancy or breastfeeding, presence of chronic inflammatory diseases such as arthritis, colitis, infectious disease such as tuberculosis, in use of anti-inflammatories for more than five days in the last three months, cancer, previous pancreatitis, hypersensitivity to rhein, or severe liver or gastrointestinal disease.

Interventions

There will be two intervention groups:

Experimental group 1: 36 patients with diabetes and chronic renal disease who will receive 1 capsule of diacerein, 50 mg twice daily, orally for 90 days.

Control group: 36 patients with diabetes and chronic renal disease who will receive 1 oral capsule twice daily, orally for 90 days.

In addition to the baseline visit, participants will be assessed on visits at 7, 30, 60 and 90 days after the randomization. All participants will receive general recommendations on health care, to maintain body weight, not modify anti-diabetic treatment, to adhere to anti-diabetic treatment and the study drug.

Random allocation and allocation concealment

The randomization code will be prepared before the trial starts. The randomization list will be generated by a computer system with validated software and provided by a researcher other than those involved in the research protocol implementation and care of patients. The study drug and placebo will be dispensed by the Research Center pharmacist who will not have direct contact with the research team and will not be part of the patient care team.

Primary clinical outcomes

The primary outcomes are a 15% reduction in ACR, any reduction in GFR, reduction in plasma levels of IL-1β, IL-6, IL-8, and TNF-α, and increase in plasma IL-10 levels.

Secondary clinical outcomes

Improvement of metabolic control (defined as A1C <7% and fasting blood glucose <126 mg/dL) and reduction of blood pressure. In addition to reduction of serum levels of fasting insulin, adiponectin, leptin and selectin.

Sample size calculation

The sample size calculation was estimated, since there is no previous data on the reduction of urinary AC ratio in patients with diabetic nephropathy using diacerein. The standard deviations (SD) of the GFR for patients with mild (30 mg/g) and moderate (45 mg/g) ND were used as estimates of the SD of the urinary A/C ratio for both groups. The sample size was calculated for reductions in the intervention group compared to placebo ranging from 20 to 40 mg/g. Approximately 72 participants would be required to detect an ACR reduction of at least 20 mg/g ± 30 mg/g up to 30 mg/g ± 45 mg/g in participants using diacerein, compared to placebo, with 80% power, and P (Alpha) of 0.05, for a 1: 1 ratio of intervention to control.

Statistical analysis

Quantitative variables will be analyzed using Student t-test for comparison of means, while the Pearson chi-square test will be used for categorical data analysis. If the groups do not turn out to be homogeneous, Poisson regression models and ANCOVA can be used to control confounding factors. The analysis aims to compare whether the variation of parameters between the baseline and the end of the trial in each group differ significantly among groups at the end of the trial, for a

P value less than 0.05. If the analysis shows an effect on the metabolic control, relative risk and relative risk reduction can be calculated using Poisson regression and controlling for baseline values. Analyzes will be performed with SPSS for Windows software (version 17; SPSS Inc., Chicago, Illinois, USA). The analysis will be performed by intention to treat.

Implementation of the study

Blood samples will be collected at the randomization visit and at the 90-day visit. The laboratory analysis will be carried out in the São Vicente de Paulo Hospital and Hospital de Clínicas of Porto Alegre.

Glomerular filtration rate will be calculated by the MDRD and CKD-EPI formulas, glycosylated hemoglobin will be determined by HPLC, nephelometry microalbuminuria, lipid profile by automated enzymatic methods and LDL cholesterol fraction will be calculated by the Friedewald equation, creatinine by Jaffé reaction, and inflammatory cytokines by Luminex method. Blood pressure will be assessed through 24-hour ambulatory blood pressure monitoring (MAP-24h) using CARDIOS monitor.

Adverse events

They will be investigated through a structured questionnaire applied throughout the follow-up, including general symptoms and presumed adverse effects of the drug used in the study.

ETHICAL APPROVAL

The project was approved by the Research Ethics Committee of the Hospital de Clínicas of Porto Alegre, which is accredited by the Office of Human Research Protections as an Institutional Review Board. Participants were informed and those who agreed to participate signed the informed consent form. The protocol was approved prior to the start of the trial under the GPPG number: 120482.

REFERENCES

1.American Diabetes Association. Diagnosis and classification of diabetes mellitus. Diabetes Care. 2012;35 Suppl 1:S64-71.

2.Shaw JE, Sicree RA, Zimmet PZ. Global estimates of the prevalence of diabetes for 2010 and 2030. Diabetes Res Clin Pract. 2010;87:4-14.

3. Wild S, Roglic G, Green A, Sicree R, King H.Global prevalence of diabetes: estimates for the year 2000 and projections for 2030. Diabetes Care 2004;27:1047-53.

4.Late Complications Of Diabetes. Chapter 334: Diabetes Mellitus. Harrison's Online. Editors, Anthony S. Fauci, Eugene Braunwald, Kurt J. Isselbacher, Dennis L. Kasper, Stephen L. Hauser, and Dan L. Longo along with J. Larry Jameson, in Harrison's Online 2001 The McGraw-Hill Companies.

5.Expert Committee on the Diagnosis and Classification of Diabetes Mellitus. Report of the expert committee on the diagnosis and classification of diabetes mellitus. Diabetes Care. 2003;26 Suppl:S5-20.

6.Adler AI, Stevens RJ, Manley SE, Bilous RW, Cull CA, Holman RR; UKPDS GROUP. Development and progression of nephropathy in type 2 diabetes: the United Kingdom Prospective Diabetes Study (UKPDS 64). Kidney Int. 2003;63:225-32.

7. Gross JL, de Azevedo MJ, Silveiro SP, Canani LH, Caramori ML, Zelmanovitz T. Diabetic nephropathy: diagnosis, prevention, and treatment. Diabetes Care. 2005;28:164-76.

8.National Kidney Foundation. K/DOQI clinical practice guidelines for chronic kidney disease: evaluation, classification, and stratification. Am J Kidney Dis 2002;39:S1-266.

9.Navarro-González JF, Mora-Fernández C. The role of inflammatory cytokines in diabetic nephropathy. J Am Soc Nephrol. 2008;19:433-42.

10. Navarro-González JF, Mora-Fernández C, Muros de Fuentes M, García-Pérez J. Inflammatory molecules and pathways in the pathogenesis of diabetic nephropathy. Nat Rev Nephrol. 2011;7:327-40.

11. Lewis EJ, Greene T, Spitalewiz S, Blumenthal S, Berl T, Hunsicker LG, Pohl MA, Rohde RD, Raz I, Yerushalmy Y, Yagil Y, Herskovits T, Atkins RC, Reutens AT, Packham DK, Lewis JB; Collaborative Study Group. Pyridorin in type 2 diabetic nephropathy. J Am Soc Nephrol. 2012;23:131-36.

12.Packham DK, Wolfe R, Reutens AT, Berl T, Heerspink HL, Rohde R, Ivory S, Lewis J, Raz I, Wiegmann TB, Chan JC, de Zeeuw D, Lewis EJ, Atkins RC; Collaborative Study Group. Sulodexide fails to demonstrate renoprotection in overt type 2 diabetic nephropathy. J Am Soc Nephrol. 2012;23:123-30.

13.Mann JF, Green D, Jamerson K, Ruilope LM, Kuranoff SJ, Littke T, Viberti G; ASCEND Study Group. Avosentan for overt diabetic nephropathy. J Am Soc Nephrol. 2010;21:527-35.
14.Ramos-Zavala MG, González-Ortiz M, Martínez-Abundis E, Robles-Cervantes JA, González-López R, Santiago-Hernández NJ. Effect of diacerein on insulin secretion and metabolic control in drug-naive patients with type 2 diabetes: a randomized clinical trial. Diabetes Care. 2011;34:1591-94.
15.Tobar N, Oliveira AG, Guadagnini D, Bagarolli RA, Rocha GZ, Araujo TG, Prada PO, Saad MJ. Comment on: Ramos-Zavala et al. Effect of diacerein on insulin secretion and metabolic control in drug-naïve patients with type 2 diabetes: a randomized clinical trial. Diabetes Care 2012;35:e13.
