# Supplementary material for: Effect of diacerein on renal function and inflammatory cytokines in participants with type 2 diabetes mellitus and chronic kidney disease: A randomized controlled trial
Source: PLoS One. 2017 Oct 19;12(10):e0186554. doi: 10.1371/journal.pone.0186554 (PMC5648185; doi:10.1371/journal.pone.0186554)
Supplement: S2 Text — (PDF) [file pone.0186554.s004.pdf]

PROTOCOLO

EFEITO DA DIACEREÍNA SOBRE FUNÇÃO RENAL E PARÂMETROS INFLAMATÓRIOS EM PACIENTES COM DIABETTIS MELLITUS TIPO 2 COM NEFROPATIA DIABÉTICA: UM ENSAIO CLÍNICO RANDOMIZADO

Authors: Fabiana Piovesan, Gláucia S. Três, Leila B. Moreira, Michael Andrades, Hugo K. Lisboa, Sandra C. Fuchs

Plataforma Brasil/Sistema Nacional de Ética em Pesquisa
CAAE: 07049612.2.0000.5327
Comitê de Ética em Pesquisa do Hospital de Clínicas de Porto Alegre
(GPPG number120482)

Instituições/ Patrocinadores
Hospital de Clinicas de Porto Alegre, Universidade Federal do Rio Grande do Sul (UFRGS), Hospital São Vicente de Paulo, CNPq

Condições de Saúde:
Diabetes mellitus
Nefropatia diabetica
Insuficiência renal cronica

BACKGROUND

O diabetes mellitus é uma doença crônica que pode cursar com complicações micro e macrovasculares levando a morbidade e mortalidade relevantes (1,4,5). A prevalência estimada da doença está em torno de 8,2%, com aumento previsto para aproximadamente 366 milhões, em 2030 (2,3). Nefropatia diabética (ND) é uma complicação relevante do diabetes mellitus tipo 2 (DM2), ocorrendo em aproximadamente 20 a 40% dos pacientes com diabetes, sendo a principal causa de doença renal crônica dialítica em países desenvolvidos (1). ND é determinada pelo aumento da excreção urinária de albumina (microalbuminúria ou macroalbuminúria) e/ou redução da taxa de filtração glomerular (TFG) (6,7,8).

O conhecimento da fisiopatologia da nefropatia diabética tem avançado através de análises moleculares e genéticas que apontam para o sistema imune como um dos mediadores do processo e das complicações da doença. Citocinas inflamatórias, principalmente interleucina 1 (IL-1), interleucina 6 (IL-6) e interleucina 18 (IL-18), bem como fator de necrose tumoral (TNF α) participariam do desenvolvimento e progressão da nefropatia diabética. A presença de mastócitos no interstício tubular com liberação de quimases, fator de crescimento transformador (TGF-β1), renina e TNF-α levariam a lesão estrutural renal bem como a inflamação resultando em fibrose em última análise (9,10). A despeito dos benefícios das medicações renoprotetoras, tais como inibidores da enzima conversora e bloqueador do receptor de angiotensina, a prevalência elevada de nefropatia diabética caracteriza a necessidade de novos agentes terapêuticos.

O potencial para afetar os mecanismos primários da ND levaram a identificação e testagem de novos agentes, por exemplo, para a redução da proteinúria causada pelo diabetes, tais como estatinas, pentoxifilina, micofenolato mofetil, antagonista do receptor da endotelina, inibidor de proteinaquinase C, fenofibratos e óleo de peixe (11,12,13). Um ensaio clínico randomizado realizado em indivíduos diabéticos demonstrou efeito redutor na glicemia de jejum, hemoglobina glicada e marcadores inflamatórios após a administração de diacereína, um derivado da antraquinona com propriedades antiinflamatórias (14, 15). Outras terapias focadas no processo inflamatório da nefropatia diabética parecem ser necessárias para promover remissão ou até regressão da lesão renal. Como a diacereína possui propriedades anti-inflamatórias e já mostrou efeito em pacientes com DM (14), acreditamos que devam ser investigados seu efeito em pacientes com comprometimento renal devido ao diabetes mellitus.

QUESTÕES DE PESQUISA

1.A diacereína melhora a nefropatia diabética em indivíduos portadores de diabetes mellitus tipo 2, avaliada através da razão albumina/creatinina urinária e da TFG?

2.A diacereína modifica os níveis séricos de citocinas inflamatórias em indivíduos portadores de diabetes mellitus tipo 2, avaliada através da dosagem de IL-1β, IL-6, IL-8, IL-10 e TNF-α?

3.A diacereína melhora o controle metabólico e a pressão arterial em indivíduos portadores de diabetes mellitus tipo 2 com nefropatia diabética?

MÉTODOS

Desenho do estudo

Ensaio clínico de tratamento, paralelo, duplo-cego, randomizado controlado, com dois braços, de fase 3.

Critérios de elegibilidade

Pacientes entre 30 e 80 anos, com diabete mellitus tipo 2, com diminuição da Taxa de Filtração Glomerular (estágios 1 e 2), hemoglobina glicada entre 7,5 e 10%; em uso regular de inibidor da Enzima Conversora da Angiotensina ou bloqueador do Receptor da Angiotensina, com índice de massa corporal menor do que 40kg/m^2, fazendo uso de medicamentos antidiabéticos, que aceitem participar e assinem consentimento livre e esclarecido.

Critérios de exclusão

Mulheres gestantes ou lactantes, presença de doenças inflamatórias crônicas como artrite, colite, doença infecciosa como tuberculose, em uso de antiinflamatórios por mais do que cinco dias nos últimos três meses; diagnóstico prévio de pancreatite; câncer, hipersensibilidade a reina ou seus correlatos (laxativos), ou doença gastrointestinal grave.

Intervenções

Serão dois grupos de intervenção:

Grupo experimental 1: 36 pacientes com diabetes e doença renal cronica que receberão 1 cápsula de diacereína 50 mg duas vezes ao dia via oral por 90 dias

Grupo controle: 36 pacientes com diabetes e doença renal cronica que receberão 1 cápsula de placebo via oral duas vezes ao dia por 90 dias. Participantes do grupo placebo receberão um comprimido idêntico ao comprimido de diacerein, em formato, odor e sabor, sendo administrado da mesma maneira.

Além da consulta na linha de base, os participantes serão avaliados nas visitas realizadas em 7, 30, 60 e 90 dias após a randomização. Todos os participantes receberão recomendações gerais sobre cuidados com a saúde, para manter o peso corporal, não modificar o tratamento antidiabético, aderir ao tratamento antidiabético e ao medicamento do estudo.

Randomização e sigilo da alocação

O código de randomização será preparado antes do início do trial, sendo gerado por um sistema de computador com software validado e implementado por um pesquisador que não esteja envolvido na execução do protocolo de pesquisa ou no cuidado com os pacientes. O fármaco do estudo e o placebo serão dispensados pelo farmacêutico do Centro de Pesquisa, que não terá contato direto com a equipe de pesquisa e não fará parte da equipe responsável pelo cuidado do paciente.

Desfechos clínicos primários

Os desfechos primários são redução de 15% na ACR, qualquer redução na TFG, redução dos níveis plasmáticos de IL-1, IL-6, IL-8 e TNF-α, aumento dos níveis plasmáticos de IL-10.

Desfechos clínicos secundários

Melhora do controle metabólico (definida como A1C <7% e glicemia de jejum <126 mg / dL), redução da pressão sanguínea. Além de redução dos níveis séricos de insulina em jejum, adiponectina, leptina e selectina.

Cálculo do tamanho de amostra

O cálculo do tamanho da amostra foi baseado em estimativas, uma vez que não há dados prévios sobre a redução da razão AC urinária em pacientes com nefropatia diabética usando diacereína. Utilizaram-se os desvios padrões (DP) da TFG para ND leve (30 mg/g) e moderada (45 mg/g) como estimativa do DP da razão A/C urinaria para ambos os grupos. O tamanho da amostra foi estimado para reduções no grupo intervenção comparativamente ao placebo variando de

20 a 40 mg/g. Aproximadamente 72 participantes seriam necessários para detectar uma redução de ACR de pelo menos 20 mg/g ± 30 mg/g até 30 mg/g ± 45 mg/g em participantes que usam diacereína, comparativamente ao placebo, com 80% de potência e P (alfa) de 0,05 e uma proporção de 1: 1 de intervenção para controle.

Análise estatística

Variáveis quantitativas serão analisadas através de teste t de Student para comparação de médias, enquanto o teste do qui-quadrado de Pearson será empregado para análise de dados categóricos. Se os grupos não resultarem homogêneos, poderão ser utilizados modelos de regressão de Poisson e ANCOVA para controle de fatores de confusão. A análise pretende comparar se a variação dos parâmetros entre a linha de base e o final do ensaio em cada grupo diferem significativamente entre os grupos no final do ensaio, para um valor de P inferior a 0,05. Caso a análise mostre efeito sobre o controle metabólico, poderá ser calculado risco relativo e redução do risco relativo utilizando regressão de Poisson e controlando para valores da linha de base. Análises serão realizadas com o software SPSS for Windows (versão 17; SPSS Inc., Chicago, Illinois, USA). Análise do trial será realizada por intenção de tratar.

Implementação do estudo

Serão coletadas amostras de sangue na visita de randomização e na visita de 90 dias. Os exames serão realizados nos laboratórios de análises clínicas do Hospital São Vicente de Paulo e do Hospital de Clínicas de Porto Alegre.

Taxa de filtração glomerular será calculada pelas fórmulas de MDRD E CKD-EPI, hemoglobina glicosilada será determinada por HPLC, microalbuminúria por nefelometria, perfil lipídico por método enzimático automatizado e a fração LDL colesterol calculada pele equação de Friedewald, creatinina por reação de Jaffé e citocinas inflamatórias por método Luminex. A pressão arterial será avaliada através de monitorização ambulatorial da pressão arterial de 24h (MAPA-24h) utilizando monitor CARDIOS.

Eventos adversos

Serão investigados através de questionário estruturado aplicado em todo o seguimento, incluindo sintomas gerais e efeitos adversos presumidos da droga usada no estudo.

APROVAÇÃO ÉTICA

O projeto foi aprovado pelo Comitê de Ética em Pesquisa do Hospital de Clínicas de Porto Alegre, acreditado pelo Office of Human Research Protections como um Institutional Review Board. Os participantes foram informados e os que concordaram em participar assinaram o termo de consentimento livre e esclarecido. O protocolo foi aprovado antes do início do ensaio sob o número GPPG: 120482.

REFERENCIAS

1.American Diabetes Association. Diagnosis and classification of diabetes mellitus. Diabetes Care. 2012;35 Suppl 1:S64-71.
2.Shaw JE, Sicree RA, Zimmet PZ. Global estimates of the prevalence of diabetes for 2010 and 2030. Diabetes Res Clin Pract. 2010;87:4-14.
3. Wild S, Roglic G, Green A, Sicree R, King H.Global prevalence of diabetes: estimates for the year 2000 and projections for 2030. Diabetes Care 2004;27:1047-53.
4.Late Complications Of Diabetes. Chapter 334: Diabetes Mellitus. Harrison's Online. Editors, Anthony S. Fauci, Eugene Braunwald, Kurt J. Isselbacher, Dennis L. Kasper, Stephen L. Hauser, and Dan L. Longo along with J. Larry Jameson, in Harrison's Online 2001 The McGraw-Hill Companies.
5.Expert Committee on the Diagnosis and Classification of Diabetes Mellitus. Report of the expert committee on the diagnosis and classification of diabetes mellitus. Diabetes Care. 2003;26 Suppl:S5-20.
6.Adler AI, Stevens RJ, Manley SE, Bilous RW, Cull CA, Holman RR; UKPDS GROUP. Development and progression of nephropathy in type 2 diabetes: the United Kingdom Prospective Diabetes Study (UKPDS 64). Kidney Int. 2003;63:225-32.
7. Gross JL, de Azevedo MJ, Silveiro SP, Canani LH, Caramori ML, Zelmanovitz T. Diabetic nephropathy: diagnosis, prevention, and treatment. Diabetes Care. 2005;28:164-76.
8.National Kidney Foundation. K/DOQI clinical practice guidelines for chronic kidney disease: evaluation, classification, and stratification. Am J Kidney Dis 2002;39:S1-266.
9.Navarro-González JF, Mora-Fernández C. The role of inflammatory cytokines in diabetic nephropathy. J Am Soc Nephrol. 2008;19:433-42.
10. Navarro-González JF, Mora-Fernández C, Muros de Fuentes M, García-Pérez J. Inflammatory molecules and pathways in the pathogenesis of diabetic nephropathy. Nat Rev Nephrol. 2011;7:327-40.
11. Lewis EJ, Greene T, Spitalewiz S, Blumenthal S, Berl T, Hunsicker LG, Pohl MA, Rohde RD, Raz I, Yerushalmy Y, Yagil Y, Herskovits T, Atkins RC, Reutens AT, Packham DK, Lewis JB; Collaborative Study Group. Pyridorin in type 2 diabetic nephropathy. J Am Soc Nephrol. 2012;23:131-36.
12.Packham DK, Wolfe R, Reutens AT, Berl T, Heerspink HL, Rohde R, Ivory S, Lewis J, Raz I, Wiegmann TB, Chan JC, de Zeeuw D, Lewis EJ, Atkins RC; Collaborative Study Group. Sulodexide fails to demonstrate renoprotection in overt type 2 diabetic nephropathy. J Am Soc Nephrol. 2012;23:123-30.
13.Mann JF, Green D, Jamerson K, Ruilope LM, Kuranoff SJ, Littke T, Viberti G; ASCEND Study Group. Avosentan for overt diabetic nephropathy. J Am Soc Nephrol. 2010;21:527-35.
14.Ramos-Zavala MG, González-Ortiz M, Martínez-Abundis E, Robles-Cervantes JA, González-López R, Santiago-Hernández NJ. Effect of diacerein on insulin secretion and metabolic control in drug-naive patients with type 2 diabetes: a randomized clinical trial. Diabetes Care. 2011;34:1591-94.
15.Tobar N, Oliveira AG, Guadagnini D, Bagarolli RA, Rocha GZ, Araujo TG, Prada PO, Saad MJ. Comment on: Ramos-Zavala et al. Effect of diacerein on insulin secretion and metabolic control in drug-naïve patients with type 2 diabetes: a randomized clinical trial. Diabetes Care 2012;35:e13.
